# Metal-Free Phosphated Mesoporous SiO_2_ as Catalyst for the Low-Temperature Conversion of SO_2_ to H_2_S in Hydrogen

**DOI:** 10.3390/nano11092440

**Published:** 2021-09-18

**Authors:** Xinnan Lu, Safa Gaber, Mark A. Baker, Steven J. Hinder, Kyriaki Polychronopoulou

**Affiliations:** 1 Department of Chemical Engineering, Khalifa University, Abu Dhabi P.O. Box 127788, United Arab Emirates; 2 Department of Mechanical Engineering, Khalifa University, Abu Dhabi P.O. Box 127788, United Arab Emirates; saagaber90@gmail.com; 3 Center for Catalysis and Separations, Khalifa University, Abu Dhabi P.O. Box 127788, United Arab Emirates; 4 The Surface Analysis Laboratory, Faculty of Engineering and Physical Sciences, University of Surrey, Guildford GU2 7XH, UK; m.baker@surrey.ac.uk (M.A.B.); s.hinder@surrey.ac.uk (S.J.H.)

**Keywords:** metal-free, Si-P catalyst, SO_2_ hydrogenation, H_2_S, microwave synthesis

## Abstract

Highly active metal-free mesoporous phosphated silica was synthesized by a two-step process and used as a SO_2_ hydrogenation catalyst. With the assistance of a microwave, MCM-41 was obtained within a 10 min heating process at 180 °C, then a low ratio of P precursor was incorporated into the mesoporous silica matrix by a phosphorization step, which was accomplished in oleylamine with trioctylphosphine at 350 °C for 2 h. For benchmarking, the SiO_2_ sample without P precursor insertion and the sample with P precursor insertion into the calcined SiO_2_ were also prepared. From the microstructural analysis, it was found that the presence of CTAB surfactant was important for the incorporation of active P species, thus forming a highly dispersed, ultrafine (uf) phosphate silica, (Si-P) catalyst. The above approach led to the promising catalytic performance of uf-P@meso-SiO_2_ in the selective hydrogenation of SO_2_ to H_2_S; the latter reaction is very important in sulfur-containing gas purification. In particular, uf-P@meso-SiO_2_ exhibited activity at the temperature range between 150 and 280 °C, especially SO_2_ conversion of 94% and H_2_S selectivity of 52% at 220 °C. The importance of the CTAB surfactant can be found in stabilizing the high dispersion of ultrafine P-related species (phosphates). Intrinsic characteristics of the materials were studied using XRD, FTIR, EDX, N_2_ adsorption/desorption, TEM, and XPS to reveal the structure of the above catalysts.

## 1. Introduction

There is growing concern about the harmful effects of sulfur dioxide (SO_2_) from industrial exhaust gas on human health and the environment. Recovering sulfur from sulfur-containing gases by catalytic processes is one of the promising solutions for waste gas purification [1]. In 2019, the global market size of hydrodesulfurization catalysts was USD 2.46 billion, and between 2020 and 2027, it is projected to grow at a 4.4% annual rate. Over the last decade, rigorous environmental regulation has permitted the manufacture of low-sulfur fuels; however, there remains a huge market demand for catalysts that can be applied to convert low-sulfur-containing gases to ultralow or sulfur-free exhaust gas [2].

Currently, the commercial technologies of sulfur-containing gas hydrodesulfurization (HDS) are based on the cobalt/molybdenum sulfides supported on γ-alumina (CoMoS@Al_2_O_3_) [3,4]. Several studies have reported SO_2_ hydrogenation, in which the products are either sulfur or H_2_S. Based on the selectivity of catalysts [5,6,7,8,9], these products can be easily collected or recovered using absorption/adsorption technologies [10,11,12,13]. These SO_2_ hydrogenation processes were taken above 300 °C, which demand high energy consumption. Improving the activity of catalysts in a lower temperature range remains a challenge.

Transition metal phosphides (TMPs) have excellent capacity and good application prospects for hydroprocessing reactions. Among the TMPs, the nickel phosphide catalyst is considered the most active for hydroprocessing, even better than the commercial CoMoS@Al_2_O_3_ catalyst [14,15]. Recently, our group reported on Ni_2_P and Ni_5_P_4_ catalysts for the selective hydrogenation of SO_2_ to S and H_2_S [16,17]. By fine control of the phase, size, and morphologies of nickel phosphide, the SO_2_ hydrogenation process can take place in the temperature range of 200–300 °C. DFT studies of Ni_2_P and Ni_5_P_4_ catalysts have investigated the stability of different surfaces of nickel phosphides, which provide a deeper understanding of the adsorption and desorption of SO_2_ and H_2_ on the surface of nickel phosphide catalysts [18,19,20].

To improve catalyst performance below 200 °C, herein, we report a metal-free phosphate mesoporous silica (Si-P) catalyst for the low-temperature conversion of SO_2_ in hydrogen. By one-pot rapid microwave synthesis, mesoporous SiO_2_ with a high surface area and pore volume was developed within 15 min, then after a 2 h phosphorization step in oleylamine (OAm) and trioctylphosphine (TOP), a phospated mesoporous silica (Si-P) catalyst was obtained. This metal-free mesoporous Si-P catalyst shows an unexpected high selectivity to H_2_S and was tested for SO_2_ hydrogenation in the temperature range of 160–240 °C. The effect of silica, phosphorus, and surfactant will be discussed in what follows.

## 2. Experimental Section

### 2.1. Chemicals

Oleylamine, (OAm, ≥98%, Aldrich, St. Louis, MO, USA), trioctylphosphine (TOP, 97%, Aldrich, St. Louis, MO, USA), ethanol (≥99.8% for HPLC, Aldrich, St. Louis, MO, USA), Ludox^®^ HS-40 colloidal silica (40 wt.% in H_2_O, Sigma-Aldrich, St. Louis, MO, USA), sodium hydroxide (NaOH, 98%, Acros, Fair Lawn, NJ, USA), hexadecyl trimethyl ammonium bromide (CTAB, 98%, Sigma, St. Louis, MO, USA), and n-hexane (95% anhydrous, Aldrich, St. Louis, MO, USA) were used as received.

### 2.2. Catalyst Synthesis

**Scheme 1:**
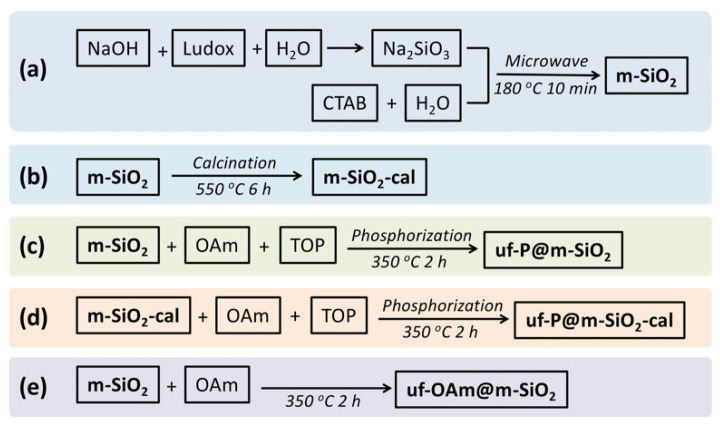
Synthetic routes indicating the preparation of the different catalysts of this study. (**a**) Microwave synthesis of m-SiO_2_ [21,22]. A 16 g amount of NaOH was dissolved in 400 mL H_2_O, then 94 mL Ludox (40 wt.%) was added to the above NaOH solution. This mixture was stirred at 40 °C in a sealed polypropylene bottle overnight until the preparation of a clear Na_2_SiO_3_ solution was achieved. A 1.0 g amount of CTAB and 35 mL H_2_O were mixed in a sealed polypropylene bottle, then the above solution was stirred slowly at 60 °C for 1 h to achieve a clear viscous solution. At the same time, 25 g of the above Na_2_SiO_3_ solution were stirred at 60 °C in a sealed polypropylene bottle for 1 h. Then, the CTAB solution was added to the Na_2_SiO_3_ solution. After 1 h of vigorous stirring at 60 °C, this mixture was removed into a 100 mL Teflon autoclave and placed in the microwave reactor (CEM MARS™ 6 Microwave Digestion and Extraction System), where a program of 5 min ramp heating to 180 °C and dwell time of 10 min at 180 °C was applied, followed by cooling to room temperature. To remove the excess of the surfactants, the solid was filtered on a Büchner funnel and washed with deionized water several times until there were no bubbles in the waste water. Then, the white solid was dried in an oven at 80 °C overnight to obtain an average of 1.5 g white powder m-SiO_2_. (**b**) Preparation of m-SiO_2_-cal [21,22]. The above-mentioned m-SiO_2_ was calcined in a tube furnace with the following stepwise process: the calcination program was set as 2 °C/min to ramp up from room temperature to 550 °C, and heating at 550 °C for 6 h, then cooled down in air. The obtained white powder was named m-SiO_2_-cal. (**c**) Preparation of uf-P@m-SiO_2_ [16,17]. In N_2_ atmosphere, 0.5 g m-SiO_2_ was first mixed with 10 mL OAm and 5 mL TOP in a three-necked flask. This mixture was under magnetic stirring for 10 min at 80 °C, then stirred at 350 °C for 2 h. These operations should be run carefully in the fume hood to avoid serious poisoning and damage to the skin and eyes caused by the TOP and its vapor. After cooling to room temperature, the powder was precipitated by centrifugation for 3 min at 4000 rpm, then washed with ethanol and hexane more than 3 times to remove the excess of the OAm and TOP on the surface of the powders. Finally, the resulting solid was dried in an oven at 80 °C overnight and named uf-P@m-SiO_2_. (**d**) Preparation of uf-P@m-SiO_2_-calIn N_2_ atmosphere. A 0.5 g amount of m-SiO_2_-cal was first mixed with 10 mL OAm and 5 mL TOP in a three-necked flask. The following procedures were the same as the preparation of uf-P@m-SiO_2_. (**e**) Preparation of uf-OAm@m-SiO_2_. In N_2_ atmosphere, 0.5 g m-SiO_2_ was first mixed with 10 mL OAm in a three-necked flask without any addition of TOP. The following procedures were the same as the preparation of uf-P@m-SiO_2_.

The systematic methodology of synthesis was followed as shown in Scheme 1, and the catalyst preparation steps involved in each sample are presented in Table 1.

### 2.3. Catalytic Assessment: Reaction Metrics 

The SO_2_ selective hydrogenation experiments were run in a fixed bed U-shaped quartz tube reactor (4 mm internal diameter) at atmospheric pressure. A 40 mg catalyst was diluted with 180 mg quartz particles (125 to 250 μm) and loaded into the reactor, followed by a pretreatment in Ar (40 mL/min) at 100 °C for 1 h. The height of the catalyst bed was about 2 cm, which was fixed by the quartz wool, and the volume of the catalyst bed was about 0.25 mL. Then, a feed gas containing SO_2_ (103 ppm, 20 mL/min) and H_2_ (20 mL/min, 2% in N_2_) were fed into the reactor. The total feed gas was set as 40 mL/min (GHSV = ~50,000 h^−1^). The reaction was run across the temperature range of 100–280 °C. The SO_2_ conversion to H_2_S for the sample was determined after 20 min on stream at 100 °C, then the temperature was increased to 120 °C and kept for 20 min. After each 20 min at a temperature platform, the temperature was set to increase by 20 °C until 280 °C. The reactants and reaction products were analyzed by a PerkinElmer Clarus 580 Gas Chromatograph (GC)-Model Arnel 4025 PPC and Model Arnel 4016 PPC Sulphur Chemiluminescence Detector (SCD) [16,17].

The conversion of SO_2_ can be described based on the following reactions:

(1) $${\mathrm{SO}}_{2}+2H_{2}\to S+2H_{2}O$$

(2) $${\mathrm{SO}}_{2}+3H_{2}\to H_{2}S+2H_{2}O$$

The yield of H_2_S, the conversion of SO_2_, and the selectivity to H_2_S were calculated based on the peak area of H_2_S and SO_2_ using the following equations:

(3) $${\mathrm{Yield}\;\mathrm{of}\;H}_{2}S\;\left(\%\right)=\frac{{\left(H_{2}S\right)}_{\mathrm{out}}}{{\left({\mathrm{SO}}_{2}\right)}_{\mathrm{in}}}\times 100$$

(4) $${\mathrm{Conversion}\;\mathrm{of}\;\mathrm{SO}}_{2}\;\left(\%\right)=\left(1-\frac{{\left({\mathrm{SO}}_{2}\right)}_{\mathrm{out}}}{{\left({\mathrm{SO}}_{2}\right)}_{\mathrm{in}}}\right)\times 100$$

(5) $${\mathrm{Selectivity}\;\mathrm{to}\;H}_{2}S\;\left(\%\right)=\frac{{\mathrm{Yield}\;\mathrm{of}\;H}_{2}S}{{\mathrm{Conversion}\;\mathrm{of}\;\mathrm{SO}}_{2}}\times 100$$

where (H_2_S)_out_ is the H_2_S molar flow rate in the exhaust gas (mol/s), (SO_2_)_in_ is the SO_2_ molar flow rate in the feed gas (mol/s), and (SO_2_)_out_ is the SO_2_ molar flow rate in the exhaust gas (mol/s).

### 2.4. Characterization of Catalyst Structure

Glancing angle X-ray powder diffraction analysis was carried out using a Rigaku Ultima IV X-ray diffractometer with the CuKα radiation (λ = 1.5406 Å, 35 kV, 25 mA). Nitrogen adsorption/desorption isotherms were recorded at 77 K using a 3Flex Micromeritics (Atlanta, GA, USA) instrument (~90 mg sample was pretreated at 100 °C in vacuum overnight for degassing). The morphologies of the catalysts were determined using a high-resolution transmission electron microscopy HR-TEM (FEI Tecnai, 200 kV). Nanoscale TEM elemental mapping was carried out using JEM-F200 electron microscope (EX-37001). Fourier transform infrared spectra were measured by a FTIR-FRONTIER/PERKIN ELMER spectrometer. Elemental analysis was conducted in an FEI Quanta 250 FEG scanning microscope (SEM). The atomic percentage (%) of the components was calculated from five measurements done at various locations/magnifications. The photoelectron spectra of the samples were carried out using a ThermoFisher Scientific k-alpha X-ray Photoelectron Spectrometer (East Grinstead, UK, monochromatic Al Kα X-ray source, hv = 1486.6 eV). The wide scan spectra were recorded using an analyzer pass energy of 300 eV. For the high-resolution core-level spectra, the pass energy was reduced to 50 eV. Quantification was performed using instrument-modified sensitivity factors following a Shirley background removal. The binding energies given are referenced to the adventitious C 1 s peak at 285.0 eV.

## 3. Results and Discussion

### 3.1. Catalyst Characterization

**Figure 1:**
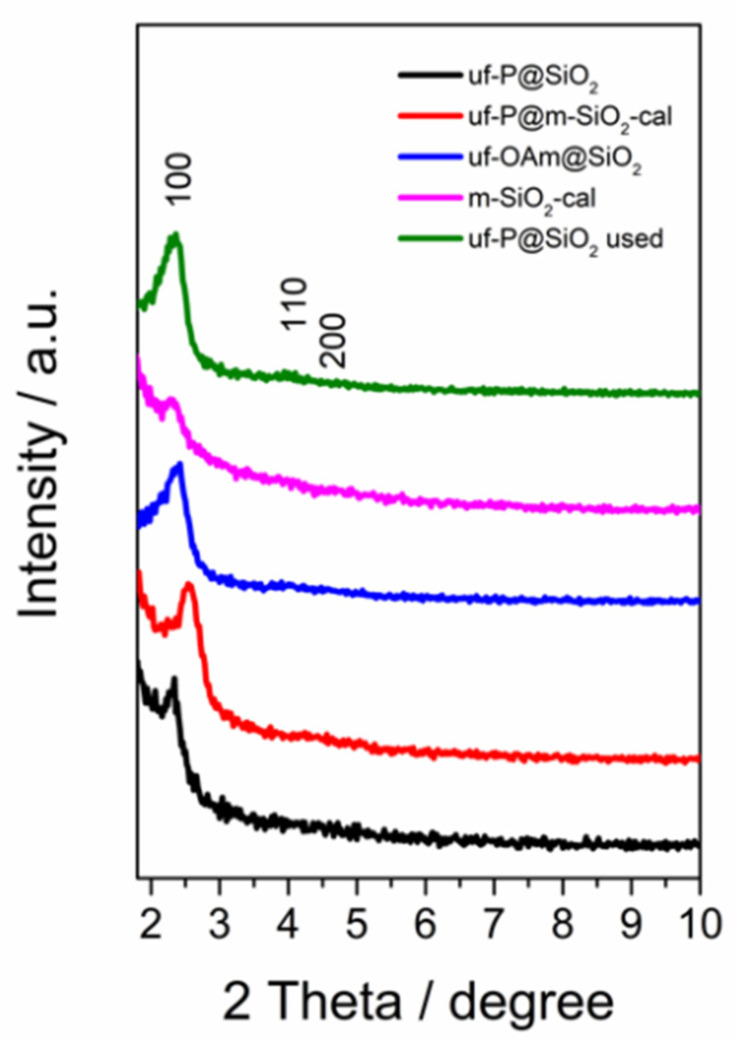
Glancing angle XRD patterns uf-P@m-SiO_2_ (fresh and used), uf-P@m-SiO_2_-cal, uf-OAm@m-SiO_2_, and m-SiO_2_-cal samples.

The low-angle XRD patterns of samples in Figure 1 display three characteristic peaks corresponding to the (100), (110), and (200) plane diffractions, respectively, typical of the hexagonal structure (p6mm symmetry) of MCM-41 [21,22]. In addition to the broad peak originating from the amorphous SiO_2_ at around 20° [23], no other obvious peaks were observed between 2θ of 10° and 70° (Figure S1), showing the absence of any crystalline phase related to P species. For the uf-P@m-SiO_2_-cal and m-SiO_2_-cal samples, the diffraction peaks decrease after the removal of the surfactant by calcination (550 °C in air), while for the uf-P@m-SiO_2_ sample, the phosphorization process (350 °C in OAm) did not lead to collapse of the hexagonal structure.

**Figure 2:**
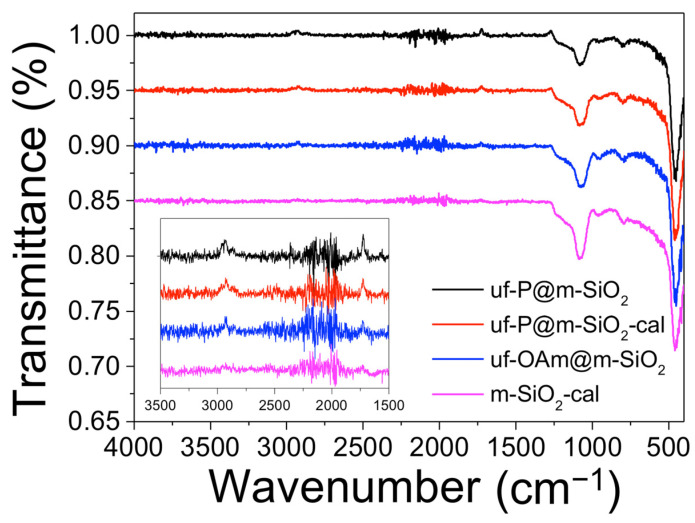
Fourier transform infrared (FTIR) spectra of uf-P@m-SiO_2_, uf-P@m-SiO_2_-cal, uf-OAm@m-SiO_2_, and m-SiO_2_-cal samples.

In Figure 2, all four samples present typical FTIR bands of the MCM-41 matrix [24]. The peaks at around 800 cm^−1^ corresponding to the Si–OH bending vibration and at around 960 cm^−1^ are the peaks of the Si-OH bond stretching vibration of silanol groups. The peaks centered at 1080 cm^−1^ and 1230 cm^−1^ correspond to the Si–O–Si asymmetric stretching vibration. The small peaks at 1725 cm^−1^ indicate the existence of –C=O stretch, which comes from the residual CTAB and OAm. The peaks at 2870 cm^−1^, 2930 cm^−1^, and 2950 cm^−1^ are associated with the –C–H stretching vibration of residual CTAB and OAm. In the 1100–1250 cm^−1^ region, the peaks related to phosphates can be found. However, due to their overlapping with other peaks and the low P precursor loading, it is rather difficult to clearly observe the peaks assigned to P-related functional groups. The phosphates origin can be due to the fact that TOP (P-bearing precursor compound) reacts at 350 °C/2 h with silica O^2−^/OH^−^ groups, part of the P-compound decomposes (carbon chain), and the P centers react with O^2−^/OH^−^ from silica forming the phosphates.

The phosphorization process took place by mixing 10 mL oleylamine and 5 mL trioctylphosphine. A 0.5 g amount of SiO_2_ powder was dispersed into the above solution. The initial atomic ratio of P to Si is between 1 and 2, and excess P compared to Si in the initial mixture was introduced to ensure the sufficient access of P to the SiO_2_ matrix. The final loading of P to Si is less than 0.1% (Table 2 and Table S1), as the P was highly dispersed onto the SiO_2_ matrix. The loading of P compared to the SiO_2_ matrix, expressed as the P/Si atomic concentration ratio and determined by SEM-EDX and XPS, is shown in Table 2, showing the P/Si atomic concentration ratio. Si in the fresh samples of uf-P@m-SiO_2_ and uf-P@m-SiO_2_-cal is about 0.4%, and 0.8%, respectively, while in the spent samples of uf-P@m-SiO_2_ and uf-P@m-SiO_2_-cal, it is about 0.4% and 1.1%, respectively. No P was found in the samples uf-OAm@m-SiO_2_ and m-SiO_2_-cal. The relatively low P content assures the high dispersion of phosphate groups.

**Figure 3:**
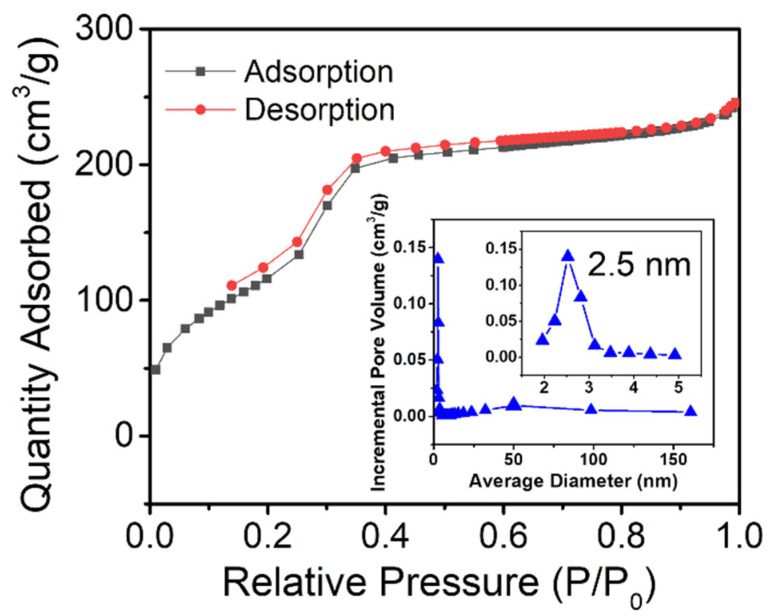
N_2_ adsorption/desorption isotherms of uf-P@m-SiO_2_ with their average pore width versus incremental pore volume, determined via the BJH method by desorption isotherms.

The textural property of uf-P@m-SiO_2_ was studied by N_2_ adsorption/desorption experiments at 77 K. The collected isotherm along with the pore size distributions are presented in Figure 3. According to the IUPAC classification, Figure 3 exhibits the typical type IV isotherm [25]. There are three different volume increases: the increases at low, medium, and high relative pressure, which correspond to the filling of microporous, mesoporous, and intergrain volumes, respectively. For uf-P@m-SiO_2_, the BET surface area is 533 m^2^/g, the pore volume is 0.38 cm^3^/g, and the BJH pore size sharp peaks are centered at 2.5 nm, which is attributed to the mesoporous channels. Compared to the surface area and pore volume of MCM-41 in the literature (~1000 m^2^/g, ~1.0 cm^3^/g) [21,22], the pores of uf-P@m-SiO_2_ are partially occupied, covered with the residual surfactant, and incorporated of P into the SiO_2_ structure, the latter being an active nonporous phase.

**Figure 4:**
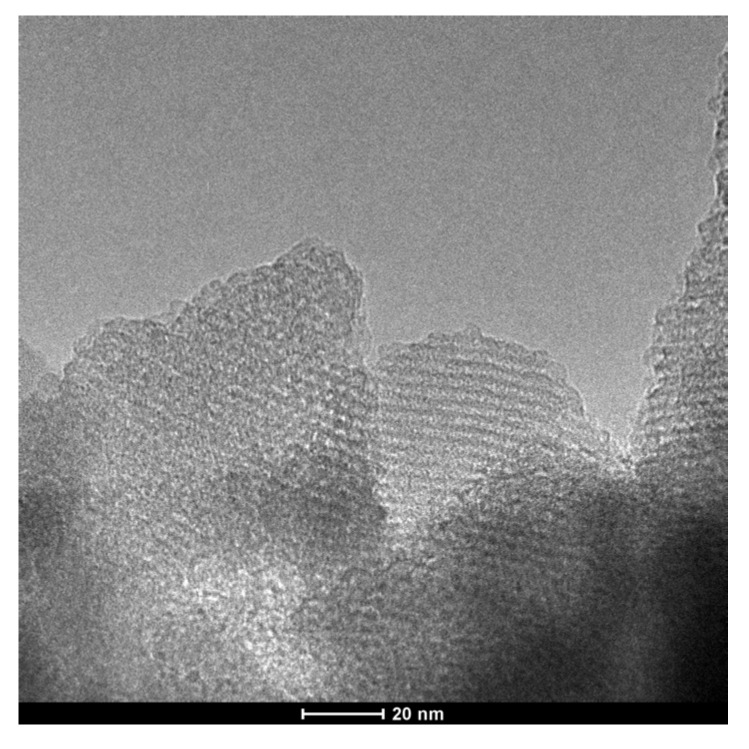
TEM image of uf-P@m-SiO_2_.

**Figure 5:**
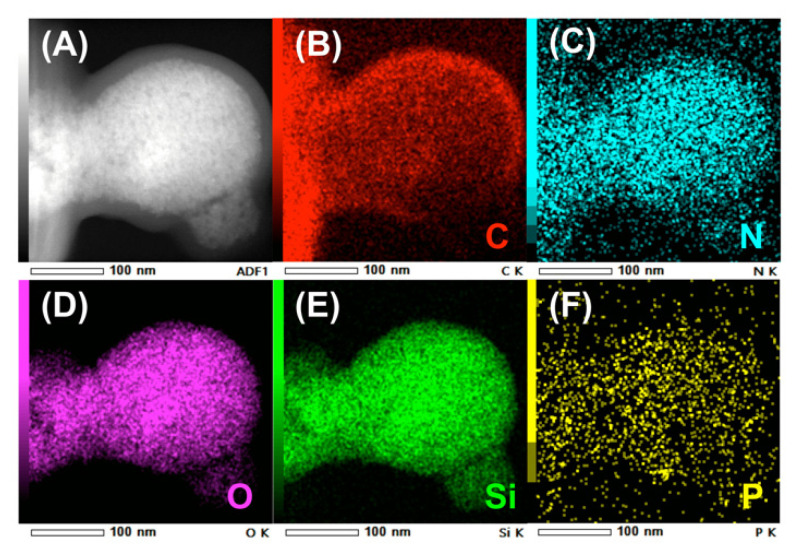
TEM elemental mapping of uf-P@m-SiO_2_. (**A**) the TEM image of uf-P@m-SiO_2_, (**B**) the C element mapping, (**C**) the N element mapping, (**D**) the O element mapping, (**E**) the Si element mapping (**F**) the P element mapping.

The TEM image of uf-P@m-SiO_2_ displays the mesoporous SiO_2_ framework (Figure 4), which indicates a typical MCM-41 structure [21,22]. The ordered fingerprint of the hexagonal honeycomb SiO_2_ skeleton can be clearly observed. The silica walls show a thickness of about 1 nm, while the pore diameter is about 2 nm. This is consistent with the average pore diameter data from the N_2_ adsorption/desorption result (Figure 3). Despite that the uf-P@m-SiO_2_ sample was synthesized following a phosphorization process at 350 °C for 2 h, there was neither obvious collapse nor destruction of the SiO_2_ matrix nor formation of a domain related to the P species. In order to track the P-related phase dispersion, TEM elemental mapping of C, N, O, Si, and P was carried out at different positions over the uf-P@m-SiO_2_ sample (Figure 5, Figures S2 and S3). Figure 5F shows that the P species were dispersed uniformly in the uf-P@m-SiO_2_ sample. In addition to Si (Figure 5E) and O (Figure 5D), which are components of the SiO_2_ framework, the rest of the elements, namely C (Figure 5B) and N (Figure 5C), are also observed, which are uniformly dispersed in uf-P@m-SiO_2_. That the source of C and N is the nondecomposed CTAB surfactant (synthesis remnant) allows us to consider that CTAB and P-related species are dispersed in the same areas. The EDX quantitative analysis of uf-P@m-SiO_2_ based on TEM mapping (Table S2, Figures S2–S4) indicates the P/Si atomic concentration ratio to be 0.4%, which is consistent with the SEM-EDX result (Table 2).

**Figure 6:**
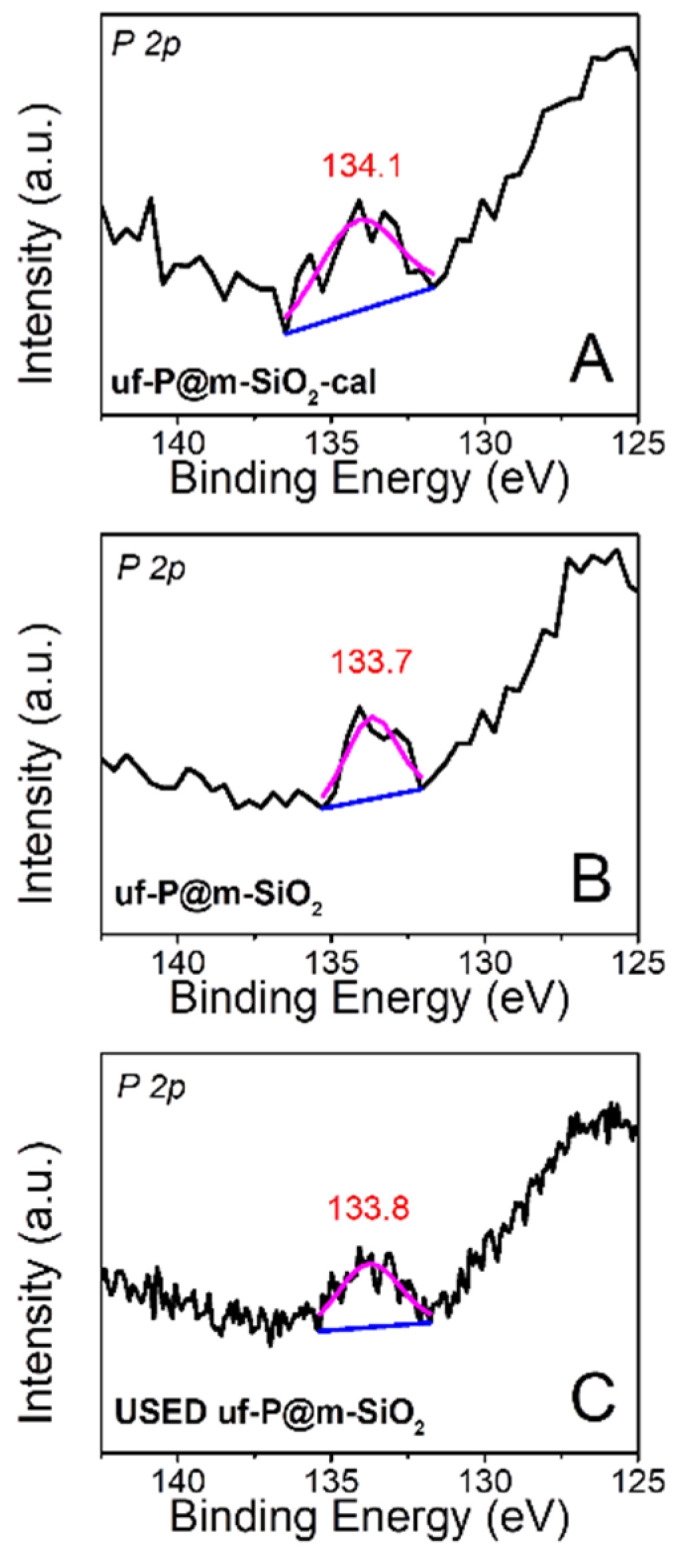
High-resolution XPS spectra of uf-P@m-SiO_2_-cal (**A**): fresh and uf-P@m-SiO_2_ (**B**); fresh and (**C**) used.

The X-ray photoelectron spectra (XPS) of uf-P@m-SiO_2_-cal (Figure 6A: fresh) and uf-P@m-SiO_2_ (Figure 6B: fresh; Figure 6C: used) were collected to identify the chemical state of P. The peak is located at ~134 eV for all samples, which can be attributed to the presence of phosphate species [26]. There is no peak at a binding energy of 130 eV, typical of elemental phosphorus or phosphorus pentoxide at 135.0–135.5 eV [26]. The XPS quantitative analysis of uf-P@m-SiO_2_ (Table S1) indicates the atomic concentration ratio of P/Si is about 0.4 at.%, which is consistent with the result from SEM-EDX (Table 2) and TEM-EDX data (Table S2). The atomic concentration ratio of P/Si and the P peaks (Figure 6) do not show obvious differences between uf-P@m-SiO_2_-cal and uf-P@m-SiO_2_ (Figure 6B: fresh; Figure 6C: used), while the C and N ratios in used uf-P@m-SiO_2_ are lower than the C and N ratio in the fresh uf-P@m-SiO_2_ sample. The latter is anticipated due to the reaction temperature and the slow decomposition of residual CTAB during the reaction.

### 3.2. Catalytic Performance

**Figure 7:**
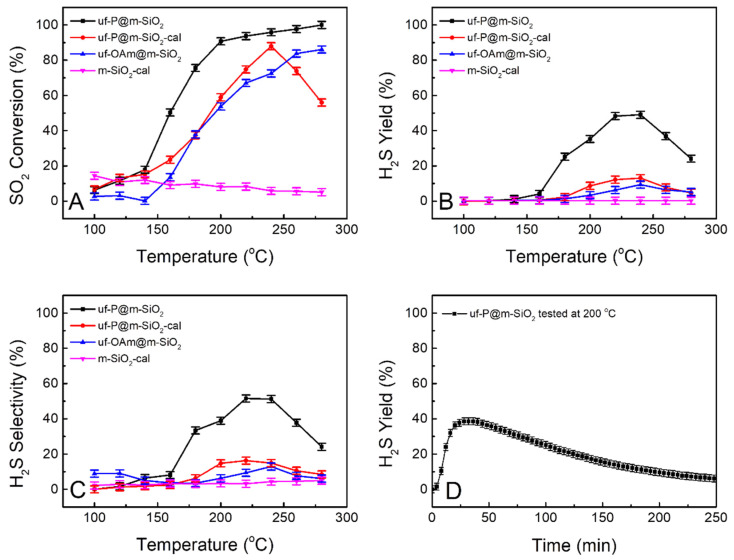
Curves of SO_2_ conversion (**A**), H_2_S yield (**B**), and H_2_S selectivity (**C**) obtained from uf-P@m-SiO_2_, uf-P@m-SiO_2_-cal, uf-OAm@m-SiO_2_, and m-SiO_2_-cal at the temperature of 100 °C to 280 °C. H_2_S yield of uf-P@m-SiO_2_ long-term test at 200 °C (**D**).

Figure 7 shows the catalytic performance test results of the four samples of interest in the present study for the conversion of SO_2_ to H_2_S in the presence of hydrogen. The pure SiO_2_ sample of m-SiO_2_-cal was tested as a reference for the other samples, and it was considered to have no activity as a blank. The other three samples show an increased conversion of SO_2_ with the increase in temperature over 150 °C (Figure 7A). The diffusion of reactants to the catalyst were negligible based on the Weisz-Prater Criterion and Mears Criterion (Table S3). Even without P, the uf-OAm@m-SiO_2_ sample shows effective SO_2_ conversion and around 9% of H_2_S yield at 240 °C (Figure 7B, blue curve). The uf-P@m-SiO_2_-cal sample has a similar H_2_S yield of around 13% at 240 °C (Figure 7B, red curve). The uf-P@m-SiO_2_ sample has a H_2_S yield of 25% at 180 °C and achieves the highest H_2_S yield of 49% at 220–240 °C (Figure 7B, black curve). Below 240 °C, the activities of the above catalysts increase with the increase in temperature, while when the temperature exceeds 240 °C, their activities begin to decrease. A long-term test was run at 200 °C for the uf-P@m-SiO_2_ catalyst (Figure 7D). The H_2_S yield reached 39% after the 30 min test and then slowly decreased. After 250 min, the H_2_S yield dropped to 6%. It is believed that phosphates act as electronegative sites, increasing the acidity of silica –OH sites. Due to the ultrafine nature of the uf-P@m-SiO_2_ sample, it is suggested that the silica originated from –OH, and the phosphates groups are adjacent. The adsorbed SO_2_ species are hydrogenated by the highly acidic H of silica, while H_2_ in the feed replenishes the OH centers in silica.

The mesoporous SiO_2_ was calcined at 550 °C for 6 h to remove the surfactant. Following this treatment, no organic residue in the mesoporous SiO_2_ was expected. This is the “m-SiO_2_-cal” sample that was run as a reference material among the four samples for the catalytic performance test. By comparison, the phosphorization processes in the solution of oleylamine and trioctylphosphine for the sample “m-SiO_2_” (as-made mesoporous SiO_2_ without calcination) and the “m-SiO_2_-cal sample” were run to achieve the “uf-P@m-SiO_2_” sample (containing organic residual template to improve the P loading) and the sample “uf-P@m-SiO_2_-cal” (without organic residual template) (Table 1). It has been reported that the active component can be highly dispersed by the assistance of the surfactant during the synthesis of a zeolite-supported catalyst [27]. We consider that the P-related phase (phosphates) dispersion was effectively influenced by the presence of surfactants, which may lead to the difference observed in the catalyst performance. It is obvious that uf-P@m-SiO_2_ shows much better catalytic performance than uf-P@m-SiO_2_-cal (Figure 7B,C). Although the P loading of both samples is similar (Table 2), the P dispersion state can be very different. During the phosphorization step, when the temperature goes above 200 °C, the TOP slowly decomposes, and the P is slowly loaded onto the SiO_2_ surface. Without the presence of the surfactant, the loading of P is similar to a conventional impregnation step, with no surface control of the P position and subsequent P aggregation, while with the assistance of the surfactant, the P site can be dispersed slowly and uniformly on the SiO_2_ matrix to avoid any aggregation, thus leading to the high population of active sites [16,27]. For the long-term test, the residual CTAB gradually decomposed during the reaction process at high temperature, resulting in the change in the dispersion state of the active phosphates component on the surface, and the activity of uf-P@m-SiO_2_ slowly decreases.

A critical comparison of the present catalysts with catalysts for SO_2_ conversion, reported already in the literature (Table S4), leads to the following conclusions. Most of the reactions in the literature were run at high temperature i.e., 240–550 °C, and the metal species are necessary as the active components (Fe/Co/Ni, etc.). In this study, no metal precursor was involved, and the P loading ratio is very low (<1%). The much lower activity temperature range herein (160 °C–240 °C) compared to the reports in the literature can fit the industry requirement of low energy cost.

## 4. Conclusions

Highly dispersed metal-free phosphated mesoporous silica was obtained with the coexistence of a CTAB surfactant in mesoporous SiO_2_ and tested as a catalyst for SO_2_ hydrogenation. This uf-P@m-SiO_2_ catalyst was applied for the low-temperature conversion of SO_2_ to H_2_S in hydrogen and showed a H_2_S yield of 49% at 220 °C. The synthesis of mesoporous SiO_2_ matrix was achieved in a 10 min heating process in a microwave at 180 °C, and the phosphorization step was completed in OAm and TOP at 350 °C for 2 h. TEM images show the hexagonal honeycomb mesopores of SiO_2_ with a silica wall thickness of 1 nm and a pore diameter of 2 nm, while N_2_ adsorption/desorption isotherms show the BET surface area of the uf-P@m-SiO_2_ catalyst is 533 m^2^/g, and the pore volume is 0.38 cm^3^/g. Elemental analysis and mapping indicated that P (about 0.4 at.% to Si) was dispersed uniformly on the SiO_2_ framework. The residual undecomposed CTAB is a key factor for the high dispersion of active P species (phosphates). The synthesis process is simple and fast, without the introduction of expensive metal precursors, and the product was easy to separate. This metal-free phosphated mesoporous silica catalyst can inspire the design and synthesis of highly dispersed active catalysts containing phosphate groups for a variety of hydrogenation applications.

## Figures and Tables

**Table 1:** Catalyst preparation steps based on the synthesis procedures followed.

Catalyst Name	SiO_2_	Calcination	Phosphorization
uf-P@m-SiO_2_	Yes	No	Yes
uf-P@m-SiO_2_-cal	Yes	Yes	Yes
uf-OAm@m-SiO_2_	Yes	No	No
m-SiO_2_-cal	Yes	Yes	No

**Table 2:** SEM-EDX and XPS determined P/Si atomic concentration ratio of the catalysts before and after the catalytic performance test.

Catalyst	EDX		XPS	
	P/Si (Atomic Concentration Ratio)			
	Fresh	Spent	Fresh	Spent
uf-P@m-SiO_2_	0.004	0.004	0.004	0.004
uf-P@m-SiO_2_-cal	0.008	0.011	0.004	/
uf-OAm@m-SiO_2_	0.0	0.0	/	/
m-SiO_2_-cal	0.0	0.0	/	/

## Data Availability

Not applicable.

## Supplementary Materials

The following are available online at https://www.mdpi.com/article/10.3390/nano11092440/s1, Figure S1: XRD patterns uf-P@m-SiO_2_ (fresh and used), uf-P@m-SiO_2_-cal, uf-OAm@m-SiO_2_, and m-SiO_2_-cal, Figure S2: TEM elemental mapping of uf-P@m-SiO_2_ (Position 2). (A,B) the TEM image of uf-P@m-SiO_2_, (C) the C element mapping, (D) the N element mapping, (E) the O element mapping, (F) the Si element mapping (G) the P element mapping, Figure S3: TEM elemental mapping of uf-P@m-SiO_2_ (Position 3). (A,B) the TEM image of uf-P@m-SiO_2_, (C) the C element mapping, (D) the N element mapping, (E) the O element mapping, (F) the Si element mapping (G) the P element mapping, Figure S4: TEM2-EDX total area spectrum recorded from the mapped area in Figure 5, Figures S2 and S3 for the sample uf-P@m-SiO_2_, Table S1: XPS elemental concentrations for uf-P@m-SiO_2_-cal (A: fresh), and uf-P@m-SiO_2_ (B: fresh, and C: used), Table S2: TEM-EDX elemental concentrations for uf-P@m-SiO_2_, Table S3: Weisz-Prater Criterion and Mears Criterion calculation for the catalyst performance test, Table S4: Comparison of the catalytic performance of different catalysts from literature of the SO_2_ conversion to H_2_S, selectivity and yield of H_2_S.
