# Supplementary material for: Metal-Free Phosphated Mesoporous SiO2 as Catalyst for the Low-Temperature Conversion of SO2 to H2S in Hydrogen
Source: Nanomaterials (Basel). 2021 Sep 18;11(9):2440. doi: 10.3390/nano11092440 (PMC8469184; doi:10.3390/nano11092440)
Supplement: Supplementary file 1 [file nanomaterials-11-02440-s001.zip › nanomaterials-1350300-supplementary.pdf]

nanomaterials

SUPPORTING INFORMATION

Metal-Free Phosphated Mesoporous SiO_2 as Catalyst for the Low-Temperature Conversion of SO_2 to H_2S in Hydrogen

Xinnan Lu [1,2,*], Safa Gaber [2,3], Mark A. Baker [4], Steven J. Hinder [4] and Kyriaki Polychronopoulou [2,3,*]

[1] Department of Chemical Engineering, Khalifa University, Abu Dhabi P.O. Box 127788, United Arab Emirates
[2] Department of Mechanical Engineering, Khalifa University, Abu Dhabi P.O. Box 127788, United Arab Emirates; saagaber90@gmail.com
[3] Center for Catalysis and Separations, Khalifa University, Abu Dhabi P.O. Box 127788, United Arab Emirates
[4] The Surface Analysis Laboratory, Faculty of Engineering and Physical Sciences, University of Surrey, Guildford GU2 7XH, UK; m.baker@surrey.ac.uk (M.A.B.); s.hinder@surrey.ac.uk (S.J.H.)
* Correspondence: xinnan.lu@ku.ac.ae (X.L.); kyriaki.polychrono@ku.ac.ae (K.P.)

Citation: Lu, X.; Gaber, S.; Baker, M.A.; Hinder. S. J.; Polychronopoulou, K. Metal-Free Phosphated Mesoporous SiO_2 as Catalyst for the Low-Temperature Conversion of SO_2 to H_2S in Hydrogen. *Nanomaterials* **2021**, *11*, 2440. https://doi.org/10.3390/nano11092440

Academic Editor(s): Detlef W. Bahnemann

Received: 05 August 2021
Accepted: 08 September 2021
Published: 18 September 2021

Publisher's Note: MDPI stays neutral with regard to jurisdictional claims in published maps and institutional affiliations.

Copyright: © 2021 by the authors. Licensee MDPI, Basel, Switzerland. This article is an open access article distributed under the terms and conditions of the Creative Commons Attribution (CC BY) license (http://creativecommons.org/licenses/by/4.0/).

Table S1. XPS elemental concentrations for uf-P@m-SiO_2-cal (A: fresh), and uf-P@m-SiO_2 (B: fresh, and C: used).

Element	Atomic %		
	uf-P@m-SiO_2-cal	uf-P@m-SiO_2 (Fresh)	uf-P@m-SiO_2 (Used)
C	20.3	20.6	16.7
N	0.9	0.9	0.7
O	53.4	53.1	56.0
Si	25.2	25.3	26.5
P	0.1	0.1	0.1

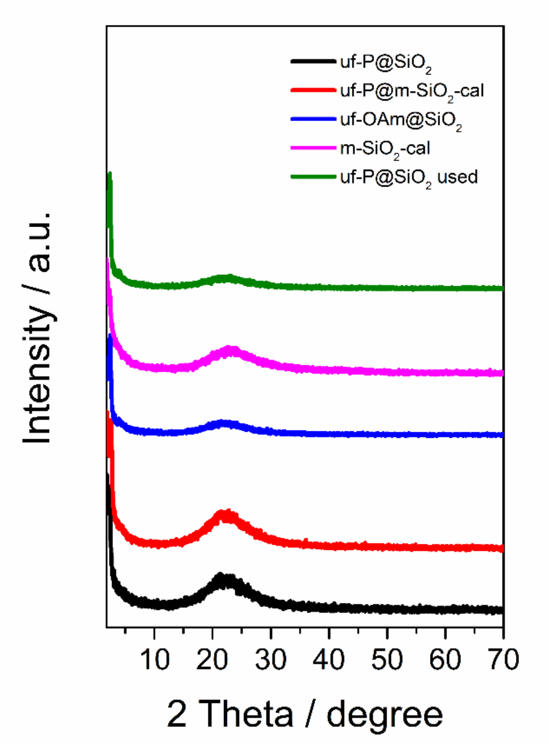

Figure S1. XRD patterns uf-P@m-SiO_2 (fresh and used), uf-P@m-SiO_2-cal, uf-OAm@m-SiO_2, and m-SiO_2-cal.

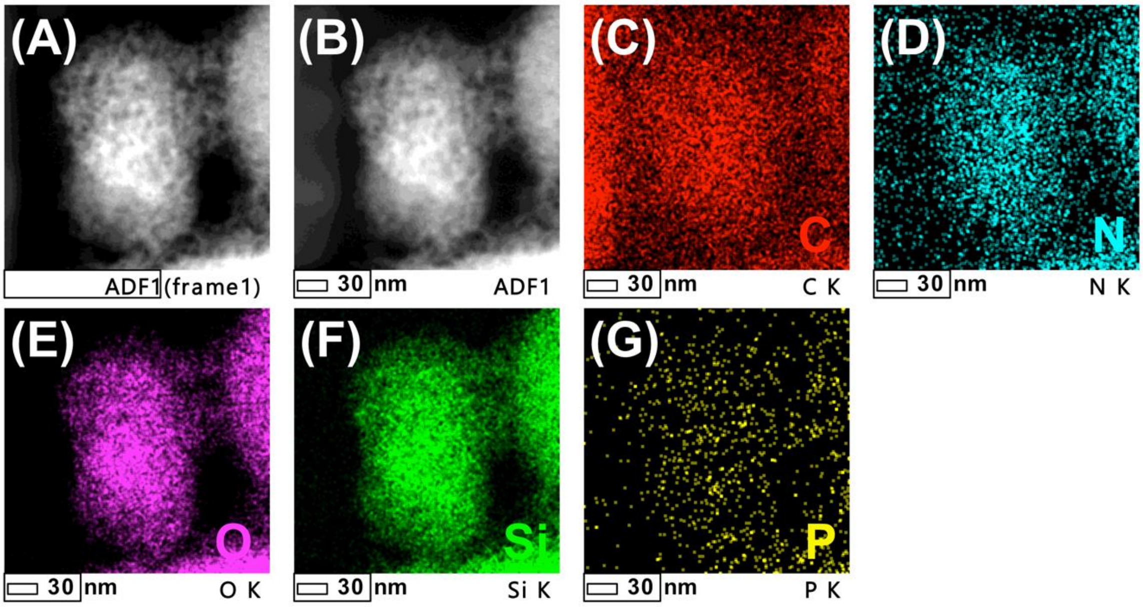

Figure S2. TEM elemental mapping of uf-P@m-SiO2 (Position 2). (**A**,**B**) the TEM image of uf-P@m-SiO2, (**C**) the C element mapping, (**D**) the N element mapping, (**E**) the O element mapping, (**F**) the Si element mapping (**G**) the P element mapping.

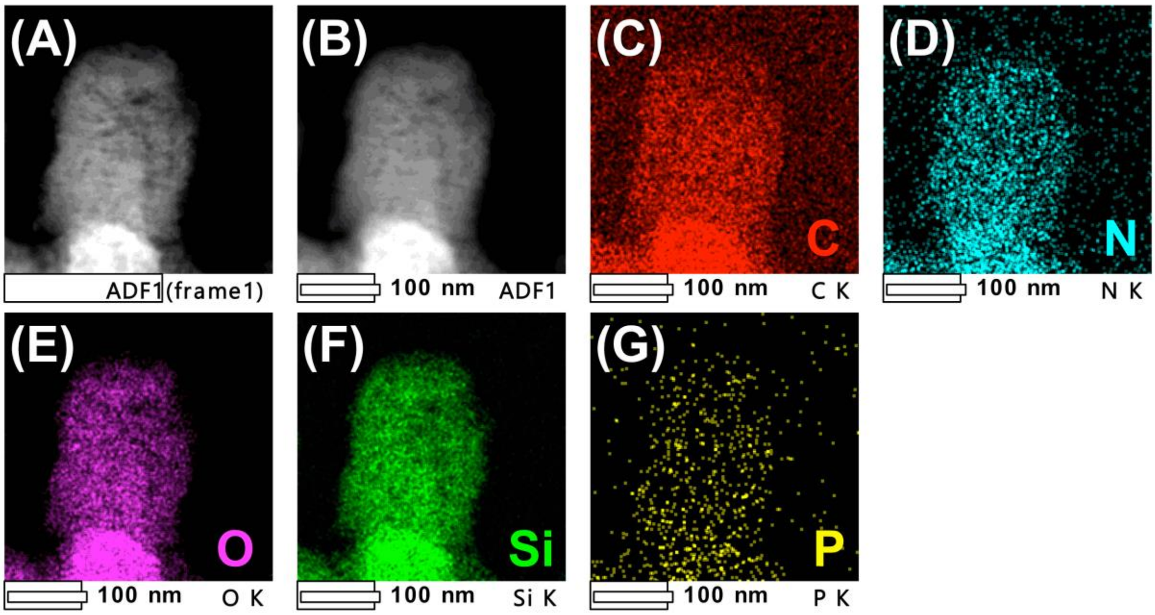

Figure S3. TEM elemental mapping of uf-P@m-SiO2 (Position 3). (**A**,**B**) the TEM image of uf-P@m-SiO2, (**C**) the C element mapping, (**D**) the N element mapping, (**E**) the O element mapping, (**F**) the Si element mapping (**G**) the P element mapping.

Position 1. (corresponding to Figure 5)

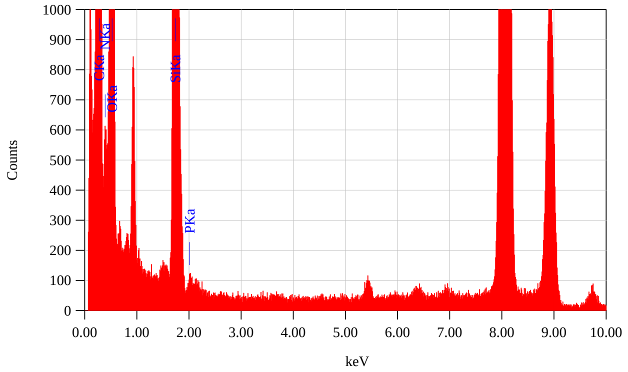

Position 2. (corresponding to Figure S2)

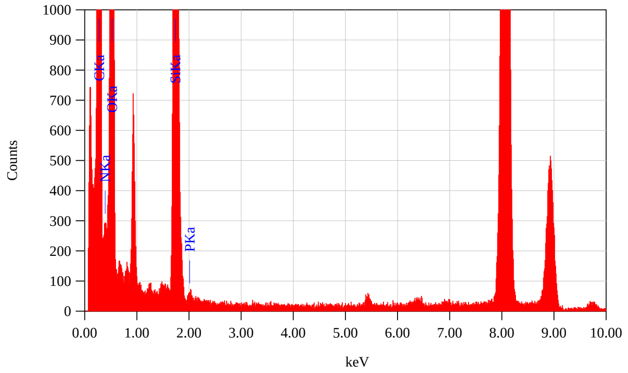

Position 3. (corresponding to Figure S3)

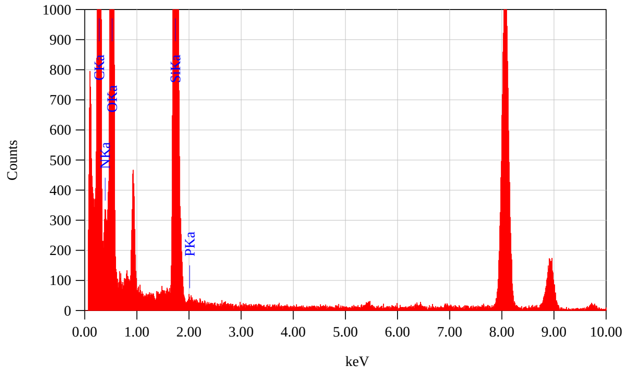

Figure S4. TEM-EDX total area spectrum recorded from the mapped area in Figure 5, Figure S2, and Figure S3 for the sample uf-P@m-SiO_2.

Table S2. TEM-EDX elemental concentrations for uf-P@m-SiO_2.

Element	Atomic %		
	**Position 1**	**Position 2**	**Position 3**
C	74.97	70.53	67.31
N	0.60	0.51	0.68
O	16.83	19.87	22.41
Si	7.57	9.05	9.58
P	0.03	0.03	0.03

Table S3. Weisz-Prater Criterion and Mears Criterion calculation for the catalyst performance test.

**Weisz-Prater Criterion**	
R (Catalyst particle radius, cm)	2.0E-02
ρ_c (catalyst density, g cm^{-3})	1.6E-02
C_{As} (Gas concentration at the catalyst surface, mol cm^{-3})	4.5E-05
D_c (Effective diffusivity cm^2 s^{-1})	1.0E-05
$r_{A\ obs}$ (mol g^{-1} s^{-1})	1.0E-07
C_{wp}	1.4E-02
**Mears Criterion**	
n (reaction order)	1
R (Catalyst particle radius, cm)	2.0E-02
ρb (bulk catalyst density, g cm^{-3})	8.7E-01
k c (mass transfer coefficient cm s^{-1})	5.3
C_{Ab} (bulk gas concentration mol cm^{-3})	4.5E-05
rA_{obs} (mol g^{-1} s^{-1})	1.0E-07
C_M	7.4E-06

The Weisz-Prater Criterion is far below 1, and Mears Criterion is far below 0.15, so the diffusion of reactants to the catalyst were negligible. [16, 28-29]

Table S4. Comparison of the catalytic performance of different catalysts from literature of the SO_2 conversion to H_2S, selectivity and yield of H_2S.

Catalyst	T (°C)	SO_2 Conv. (%)	H_2S Select. (%)	H_2S Yield (%)	Reference
**NiO/r-Al_2O_3**	320	98	31	30.4	[30]
**SnO_2-ZrO_2**	550	98	45	44.1	[31]
**Fe/γ-Al_2O_3**	240	100	100	100	[9]
**Fe-Si-Cr**	190	100	100	100	[32]
**l-Ni_5P_4@SiO_2**	325	96	99	95	[17]
**u-Ni_5P_4@SiO_2-l**	240	97	34	33	[17]
**uf-P@m-SiO_2**	140	18	6	1	This work
**uf-P@m-SiO_2**	160	50	8	4	This work
**uf-P@m-SiO_2**	180	76	33	25	This work
**uf-P@m-SiO_2**	200	91	39	35	This work
**uf-P@m-SiO_2**	220	94	52	49	This work
**uf-P@m-SiO_2**	240	96	52	50	This work

References

(1) Lu, X.; Baker, M. A.; Anjum, D. H.; et al. Ni2P Nanoparticles Embedded in Mesoporous SiO2 for Catalytic Hydrogenation of SO2 to Elemental S. *ACS Appl. Nano Mater.*, **2021**.

(2) Basina, G.; Polychronopoulou, K.; Zedan, A. F.; Dimos, K.; Katsiotis, M. S.; Fotopoulos, A. P.; Ismail, I.; Tzitzios, V. Ultrasmall Metal-Doped CeO2 Nanoparticles for Low-Temperature CO Oxidation. ACS Appl. Nano. Mater., 2020, 3, 10805-10813.

(3) Usman, M.; Li, D.; Li, C.; Zhang, S. Highly selective and stable hydrogenation of heavy aromatic-naphthalene over transition metal phosphides. Sci. China Chem., 2015, 58, 738-746.
